# Bioprocessing Data for the Production of Marine Enzymes

**DOI:** 10.3390/md8041323

**Published:** 2010-04-19

**Authors:** Sreyashi Sarkar, Arnab Pramanik, Anindita Mitra, Joydeep Mukherjee

**Affiliations:** School of Environmental Studies, Jadavpur University, Kolkata 700 032, India; E-Mails: sreyoshi_sarkar@yahoo.co.in (S.S.); arnab.ju9@gmail.com (A.P.); mitranindita@gmail.com (A.M.)

**Keywords:** marine, enzyme, bioprocess, bioreactor

## Abstract

This review is a synopsis of different bioprocess engineering approaches adopted for the production of marine enzymes. Three major modes of operation: batch, fed-batch and continuous have been used for production of enzymes (such as protease, chitinase, agarase, peroxidase) mainly from marine bacteria and fungi on a laboratory bioreactor and pilot plant scales. Submerged, immobilized and solid-state processes in batch mode were widely employed. The fed-batch process was also applied in several bioprocesses. Continuous processes with suspended cells as well as with immobilized cells have been used. Investigations in shake flasks were conducted with the prospect of large-scale processing in reactors.

## 1. Introduction

Although it has been realized that the marine realm is a rich and a largely untapped resource of products that are of potential interest to mankind, only a few of these marine natural products have reached the stage of commercial production. This gap between discovery and commercialization can be bridged when biologists, chemists and engineers join forces and integrate their research disciplines in order to develop bioprocess technologies for the production of marine natural compounds [1]. Against this backdrop, two major international symposia have been held: the first, “Marine Bioprocess Engineering”, held in Noordwijkerhout, The Netherlands, in November 1998 and the second, “Marine Biotechnology: Basics and Applications” held in Matalascañas, Spain, in February–March 2003. The first symposium showed that the biotechnological potential of the sea has been recognized not only by scientists, but also at an industrial and governmental level. Marine bioprocess engineering turned out to be a diverse and dynamic field of science, offering many possibilities to explore valuable marine resources. The second symposium focused on the relation between applications of marine biotechnology and the basic disciplines, *i.e.*, screening, chemistry, genomics, biology, process engineering and product marketing. The organizers (The European Society for Marine Biotechnology) composed a program in which both basics and applications were covered and the conference was a platform for valuable discussion between industry and academia.

A marine enzyme is a unique protein molecule with novel properties derived from an organism whose natural habitat comprises saline or brackish water. Apart from microorganisms like bacteria (including actinomycetes) and fungi, many other marine organisms such as fishes, prawns, crabs, snakes, plants and algae were also studied to tap the arsenal of the marine world. Properties like high salt tolerance, hyperthermostability, barophilicity, cold adaptivity and ease in large scale cultivation were the key interests of the scientists. These properties may not be expected in terrestrial sources as marine organisms thrive in habitats such as hydrothermal vents, oceanic caves and some areas where high pressure and absence of light is obvious [2]. A few marine-derived enzymes have reached the market, an example being Zonase X™, the leading product of Aqua Biotechnology (http://aquabiotechnology.com). Zonase X™, derived from the hatching fluid of salmon, has the normal biological function of degrading the eggshell surrounding the fish larvae during early embroyogenesis. As a skin-care product, it gently removes the dead cells in the outer layer of the human skin, initiating and enhancing the renewal and healing process of the skin. Notwithstanding this success, there is still a lack of research into bioreactor engineering and bioprocess design in the area of cultivation of marine organisms to produce enzymes as noted in the review [3]. In this article, the authors presented a summary on the successfully applied culture conditions, including media and bioprocess engineering data, first, in relation to the different biodiversities of marine prokaryotes and fungi, and second, with respect to their product classes (low and high molecular weight substances). As a part of the review, the authors also discussed marine enzyme production in relation to marine psychrophilic/psychrotrophic microbes.

Batch growth refers to culturing in a vessel with an initial charge of medium that is not altered by further nutrient addition or removal. This form of cultivation is simple and widely used both in the laboratory and industrially. Growth, product formation and substrate utilization terminate after a certain time interval, whereas in continuous culture, fresh nutrient medium is continually added to a well-stirred culture and products and cells are simultaneously withdrawn. Growth and product formation can be maintained for prolonged periods in continuous culture. After a certain period of time, the system usually reaches a steady state where cell, product and substrate concentrations remain constant. Continuous culture provides constant reactor conditions for growth and product formation and supplies uniform-quality products. In fed-batch culture, nutrients are continuously or semi-continuously fed, while effluent is removed discontinuously. Fed-batch operation permits the substrate concentration to be maintained at some pre-determined level. During substrate feeding, the reactor volume increases. At some point, part of the reactor volume is withdrawn and the process is repeated. This type of operation is intermediate between batch and continuous processes, increasing the duration of batch cultivation and the overall reactor productivity [4,5].

The performances of a culture (microbial, animal or plant cell) producing a bioactive compound is very different in a 250 mL shake flask, a 10 L laboratory reactor and a 10,000 L industrial reactor. This is due to difficulty in maintaining homogeneity in large systems, changes in surface to volume ratio and alteration in the cultures themselves due to increased length of residence time. One of the first decisions is whether to use a batch, fed-batch or continuous bioprocess. Considering productivity, continuous systems have a significant advantage when producing primary metabolites (metabolites directly involved in the normal growth, development, and reproduction), while batch systems are more suitable for secondary metabolites (chemical compounds not normally involved in primary metabolic processes such as photosynthesis and cell respiration). Genetic instability of the producer organism is higher in the continuous than in the batch mode. Batch cultures on the other hand suffer great variability from one run to another. Another factor determining reactor choice is market economics. A continuous system is dedicated to a single product, whereas batch systems offer greater flexibility. The fed-batch process is usually used to overcome substrate inhibition or catabolite repression by intermittent feeding of the substrate [4].

This review describes the different bioprocess engineering approaches adopted for the production of marine enzymes derived mainly from microbial and to a lesser extent plant or animal sources. By referring to the United States Patent database (the freely accessible part), this review focuses on enzyme production on a wider spectrum compared to Lang *et al.* [3]. The relevant bibliography has been classified based on basic bioprocess operations: batch, fed-batch and continuous processes. A separate section has been assigned to experiments done in the shake flasks with the objective of future mass production. Interesting examples of extremophiles that produce unique biocatalysts, which function under extreme conditions comparable to those prevailing in various industrial processes [6] are also provided.

## 2. Batch Processes

This segment has been divided into three major sections depending upon the basic method of cell cultivation. These sections are (1) submerged processes, where the organism is grown in a liquid medium, which is aerated and agitated in large tanks called bioreactors (for aerobic cultures) or fermenters (for anaerobic cultures) (2) immobilized systems in which the producing cell is restricted in a fixed space and (3) solid-state cultivations in which the bioprocess is operated at low moisture levels or water activities. Table 1 provides an overview on batch operating conditions while the subsequent sections provide information on novelty of the process, its merits and demerits.

### 2.1. Submerged processes

In this process, after seeding a liquid medium with an inoculum of living cells, nothing (except possibly some gas) is added to the culture or removed from it as growth proceeds. Typically in such a reactor, the concentrations of nutrients, cells and products vary with time as growth proceeds. As the submerged process is the most widely used industrial process, this section is further classified based on the type of the enzyme produced by this bioprocess.

#### 2.1.1. Proteases

The marine bacterium *Vibrio harveyi* has been used to produce extracellular proteases [7]. The bioreactor operated in the batch culture mode and was agitated with two Rushton disc turbines with two baffles. Air was sparged through a metallic tube with seven holes. Three agitation speeds (300, 500 and 700 rpm) and three air flow rates: 0.2, 0.5 and 0.8 L/L/min (litres of air per litre volume of medium per minute) were investigated. Foam was controlled by adding a few drops of sterilized sunflower oil. The lowest value of the overall oxygen mass transfer coefficient (kla termed as the product of mass transfer coefficient and interfacial area available for mass transfer) in seawater was 0.254/sec when the reactor was operated at 300 rpm and aeration of 0.2 L/L/min while the highest value recorded was 1.342/sec with agitation of 700 rpm and aeration of 0.8 L/L/min. The maximum oxygen concentration in freshwater was 18.8% higher than that in seawater, although kla values did not differ significantly. The oxygen level in seawater was sufficient to support growth, indicating that no oxygen limitation occurred due to medium salinity. Increasing agitation rates resulted in higher specific growth rate. In this study, the authors clearly demonstrated that proper selection of aeration and agitation rates resulted in high yields of protease. The effects of other reactor cultivation parameters such as medium pH, carbon sources, inducers that can influence the process performance were, however, not evaluated by the authors. In the absence of such data, bioprocess optimization remains incomplete. Kumar *et al*. [8] investigated batch cultivation of an oxidative and SDS-stable alkaline protease secreted by a marine haloalkalophilic *Bacillus clausii* (isolated from the tidal mud flats of the Korean Yellow Sea near Inchon City) under a range of process conditions. Antifoam A was used to minimize foam formation. The enzyme activity increased with an increase in process time and the rate of agitation. The increase in the protease yields may have been due to efficient mass transfer coefficients, which depend on optimal aeration and agitation rates. The major drawback of this study was that the authors did not investigate a wide range aeration and agitation rates. Like the previous report, the effects of other reactor cultivation parameters such as medium pH, carbon sources, inducers that can influence the process performance, were not evaluated by the authors. Narinx *et al*. [9] isolated the Antarctic *Bacillus* strain TA39 from sea water in the coastal area of the French Antarctic base. The production of cells at the stationary phase was half at 25 °C compared to that obtained at 4 °C whereas the protease secretion hardly reached one third of that recorded at 4 °C. After a lag phase of about 20 h, the doubling time at 4 °C during the exponential growth was 9 h compared with 2 h at 25 °C. New knowledge about subtilisin from a psychrophilic Antarctic bacterium as well as the potential application of this enzyme in commercial laundry detergents used for washing clothes in cold rather than hot water is definitely exciting. However, the major disadvantage of this process would be the high costs involved in cooling the bioreactor. Weiner *et al*. [10] described an invention related to novel extracellular alkaline metalloproteases produced by a novel marine bacterium *Hyphomonas jannaschiana* which has excellent temperature and/or pH stability. The submerged cultivation was conducted aerobically, with shaking, stirring, aerated agitation or blowing humidified air through the media by mechanical agitation or air spargers. It was possible to use any cultivation media, solid or liquid, which was suitable for conventional cultivation of microorganisms. A productive cultivation typically was in the range of 20–50 hours. Optimally, exoenzymes were detectable in the culture supernatant after about 17 hours of the start of the bioprocess. Alternatively, the cells could be harvested at the early stationary growth phase and re-suspended in a reduced volume of media to induce production of exoprotease in the culture fluid. If cell culture conditions were optimal, the cells would grow and reproduce near their theoretical maximum. The merit of this process was that a large numbers of factors affecting the bioprocess including inducers (large polypeptides) as well as end product inhibition were rigorously considered by the inventors. On the other hand, inclusion of a large number of medium components would make subsequent downstream operations more complicated.

#### 2.1.2. Chitinases

**Figure 1:**
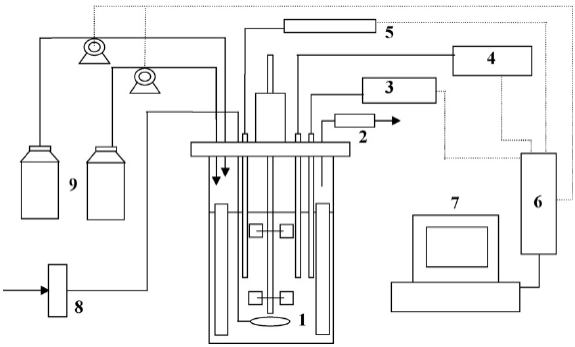
Experimental setup for production of chitinase by *Paenibacillus* sp. (1) Bioreactor; (2) exhaust air condenser; (3) pH-meter and controller; (4) DO-meter and controller; (5) thermo-meter and controller; (6) interface box; (7) computer; (8) air filter and (9) antifoam, acid, and base. Reproduced with publisher’s permission from [12].

The bioprocess conditions for optimal production of chitinolytic enzymes by *Penicillium janthineflum* (P9) were studied in a bioreactor with two agitators equipped with two standard Rushton-type turbines [11]. Silicone antifoam was added before sterilization to prevent foam formation. The influence of aeration rate and stirrer speed on the enzyme production in the bioreactor was evaluated at aeration rates of 0.5, 1.0 and 1.5 L/L/min and agitation speeds of 300, 400, 500, 600 and 700 rpm. As the differences in enzyme activity and mycelial concentration recorded between cultures grown at 24 and 28 °C were statistically insignificant, all other cultivations were carried out at 28 °C. On the industrial scale, cooling is more expensive than heating; thus, the slightly lower enzyme production at 28 °C could be compensated by the lower energy and cost required for temperature control. The microorganism probably suffered a certain degree of shear stress at stirrer speeds above 500 rpm, which resulted in lower levels of enzyme activity. Stirrer speeds lower than 400 rpm, on the other hand also resulted in decreased enzyme production, probably due to insufficient oxygenation that could be partially compensated by increasing the aeration rate. Low enzyme activity accompanied by poor mycelial growth was recorded when the fungus was grown at a stirrer speed of 300 rpm and an aeration rate of 0.5 L/L/min. After 120 hours of cultivation, the dissolved oxygen (DO) dropped to about 10% saturation and remained below that value for further 48 hours, but above 5% saturation. Although increase in agitation and aeration may provide better mixing and mass transfer effects, an excess might result in high shear stress and in turn lead to many negative effects, such as rupture of cells, vacuolation and autolysis with a consequent decrease in enzyme productivity. The optimization of the cultivation conditions led to an increase in the enzyme activity of about 65%, from 415 to 686 U/L. However, the increased chitinase production of *P. janthinellum* P9 appeared to be more dependent on appropriate combination of different process parameters than on the optimization of any single parameter which did not lead to enhancement of enzyme activity of more than 13%–20%. Kao *et al*. [12] studied the effects of operation conditions such as agitation, aeration, and pH on mass transfer and shear stress on cell morphology during chitinase production by *Paenibacillus* sp. (Figure 1).

The aeration rates varied from 1 to 3 L/L/min and the agitation rates ranged from 100 to 300 rpm. Results indicated that aeration rate directly influenced the oxygen supply, which in turn affected enzyme activity level. It was also established that oxygen transfer limitation prevailed at lower agitation rates. At an aeration rate of 3 L/L/min and agitation above 200 rpm, the Kla value reached 35.5/h. The cultivation broth was regarded as a non-Newtonian Bingham fluid (shear stress is linearly correlated to the shear rate with a slope equal to the apparent viscosity). A shear stress higher than 5.8 dyn/cm^2^ was observed to be harmful to both cell growth and chitinase production. The result presented an overall negative effect of high aeration rate on the bioprocess. The merit of this process was the comprehensive investigation on the engineering aspects (mass transfer and rheological behaviour) during chitinase production by a marine bacterium. The demerit of this process was that shear stress limited the production conditions of chitinase to certain a range of aeration and agitation. Liu *et al*. [13] established the desired combination of aeration, agitation and pH that would yield the highest chitinase activity by *Verticillium lecanii* in a stirred tank reactor (STR). The agitation rates applied were 75, 150, and 225 rpm with an aeration rate of 0.6 L/L/min. A 30 L airlift bioreactor provided with a 24-mesh net draft tube was also employed. The airflow rate varied at 0.6, 0.9, or 1.2 L/L/min. In the STR, the highest chitinase activity appeared on the sixth day after inoculation, under the 150 rpm agitation rate, thereafter, the production of chitinase decreased. The decline in chitinase production at 150 rpm agitation rate may be due to shear inactivation of the enzyme. Similar enzyme activity level appeared between the fifth and seventh day with agitation of 225 rpm, but no significant chitinase was produced with 75 rpm. The STR adapted with the baffle appeared favorable for chitinase synthesis, which meant the tank adapted with a baffle could afford better mass/gas transfer efficiency. However, the baffle also caused an aggregation of *V. lecanii* cells during the cultivation which hindered the growth of fungus and synthesis of enzyme. Although this indicated that the production of chitinase is facilitated with a certain agitation rate, the effects of agitation rate needed to be further elucidated since the biosynthesis of chitinase seemed correlated with the pH or cultivation environment. The chitinase activity changed as medium pH varied between 2 and 9 and the optimal value was determined to be 4.0. It appeared that the pH value affected the growth of *V. lecanii* as well as the release of chitinase into the medium. In the airlift bioreactor, adjustments of agitation and aeration could greatly improve mixing and gas-liquid transfer in the bioreactor. As synthesis of chitinase was related to fungal growth, an improvement on fungal growth environment would enhance chitinase synthesis. When DO concentration fell below a critical level, cell respiration shifted from DO to the gaseous form. However, this phenomenon would only occur if a high aeration rate is employed, else, a linear growth would proceed indicating a deficiency of both gaseous and DO. Results suggested that depletion of DO and associated increase in CO_2_ partial pressure could stimulate a respiration shift and a start of an altered metabolism or morphological changes. By changing agitation and aeration, oxygen supply in the bioreactor could be improved, and thus, could enhance fungal growth and enzyme production. Again, the results indicated that aeration was not the main factor affecting the production of chitinase. This study implied that the effects of pH and agitation rate on the bioprocess were more significant than aeration rate. Although the authors have noted that agitation caused morphological changes in the bioreactor, no attempt was made to alter the design of the impellers, which could reduce the effects of shear stress in the STR.

#### 2.1.3. DNA polymerases

A purified thermostable DNA polymerase was derived from the eubacterium *Thermotoga maritime* (Tma), a hyperthermophilic eubacterium that grows between 55 °C and 90 °C [14]. This eubacterium was isolated from geothermally heated sea floors in Italy and the Azores. The *Thermotoga* is a recently described genus with three recognized species, including the most extremely thermophilic eubacterium known. These organisms were originally isolated from geothermally heated marine sediments and hot springs. *E. coli* strain DG116 containing plasmid pTma12-3 encoding the *Thermotoga maritime* DNA polymerase was inoculated in a bioreactor and the culture was grown at to a cell density (A_680_) of 0.7. Foaming was controlled by the addition of propylene glycol as necessary. The growth temperature was raised from 30 °C to 35 °C to induce the synthesis of recombinant Tma DNA polymerase. The temperature shift increases the copy number of the pTma12-3 plasmid. The cells were grown for 21 hours to an optical density of 4.0 (A_680_) and harvested by centrifugation for further purification of the enzyme. Recombinant plasmid can be lost from cells due to defective segregation of plasmid during cell division or structural instability of the plasmid material due to mutations. Loss of plasmid functionality during experiments has been reported in many situations during bioreactor cultivation [15]. This could be a potential disadvantage of the process. Slater *et al*. [16] were awarded an US patent on the invention related to thermostable DNA polymerases derived from hyperthermophilic eubacteria *Thermotoga neapolitana* (Tne). For isolation of genomic DNA, large scale cultures of *T. neapolitana* cells were grown anaerobically in a defined medium in a 10 L fermentation vessel under nitrogen at 75 °C for 28 hours. The desired gene was cloned into *E. coli* strains which contained recombinant Tne (rTne) constructs. In order to produce purified preparations of the Tne polymerases, cells harbouring the Tne expression vectors were grown, induced and the Tne polymerases were isolated. This strain was used to seed 6 L of LB containing 10 μg/mL tetracycline prewarmed to 37 °C. The large scale culture was grown for 5 hours at 37 °C and then IPTG was added to a final concentration of 1 mM and growth was continued for an additional 2 hours at 37 °C. A major disadvantage of the process is the use of an expensive inducer (IPTG) which will increase the cost of the final product. The replacement of IPTG by a less expensive inducer would be desirable.

#### 2.1.4. Agarases

Agarase 0107 from the marine bacterium *Vibrio* sp. Strain JT0107 is an extremely useful enzyme for gene technology applications and for making of protoplasts of seaweeds because it exhibits optimum activity in a weakly alkaline medium and at a lower temperature relative to other enzymes reported. The production of the agarase from strain JT0107 was increased by altering culture conditions and medium composition [17]. The bioreactor was agitated at 600 rpm and aerated at various rates. In a culture with no aeration, kla was 10/h and the relative DO concentration was 0% at 5 hours of cultivation. Cell growth increased gradually and reached its maximum at 30 hours of cultivation. Though no agarase activity was observed in the first 25 hours of cultivation, 90 U/L of agarase activity was detected after 27 hours of cultivation. In an aerated culture (0.5 L/L/min), kla was 65/h, DO did not drop below 50% and the growth rate was higher than that for the culture without aeration. The maximum agarase activity of the aerated culture was approximately 7-fold higher than that of the non-aerated culture. These results indicated that the presence of dissolved oxygen significantly enhanced cell growth rate and agarase production. The overall increase by 13-fold compared to the previously reported processes is the strength of this investigation. The authors speculated enzyme induction as the reason for the drastic increase. Without a clear identification of the particular medium component as the inducer, batch-to-batch variation in enzyme levels can be expected. This may be a disadvantage of the bioprocess. The authors also obtained an US Patent for their work related to the production of novel α-agarase as well as a process for producing oligosaccharides and monosaccharides derived from agar or agarose, which have effects of preventing aging of starch or products containing starch. The enzyme was produced by *Vibrio* sp. JT0107-L4, isolated from seawater and was cultivated by a batch process in artificial sea water operated in a volume of 3 L using a 5 L jar bioreactor. Byun *et al*. [18] isolated and characterized the enzyme, arylsulfatase that showed high specificity against agar rather than other sulfated marine polysaccharides such as carrageenan, fucoidan and porphyran. Arylsulfatase hydrolyzed sulfate ester bonds in agar with out any glycosidase activity as was demonstrated by the increase of gelling strength of enzyme treated agar. The producing bacterium was identified as *Sphingomonas* sp. AS6330 as determined by 16S rDNA sequence analysis. Cell mass and enzyme yield reached their maximum after 2 days and remained at the same level for a further 2 days. This process suffers from the limitation that a wide range of cultivation parameters were not studied and optimization was not performed. An alternative strategy like continuous culture was not investigated.

#### 2.1.5. Haloperoxidases

Currently, production of marine bromoperoxidase requires field collection of macrophytic red algae from the benthic coastal marine environment. A bioprocess for bromoperoxidase production was developed [19] using *Ochtodes secundiramea* microplantlets (semi differentiated shoot tissue culture). The microplantlets were uniformly suspended by sparging humidified, filter sterilized air into the liquid medium using a porous polypropylene tube lining the bottom of the vessel. The photobioreactor was placed in a temperature-controlled incubator. The rear side of the photobioreactor was uniformly illuminated with two cool white fluorescent lamps horizontally aligned on the outer surface of the vessel. The fresh and dry cell densities were measured as a function of cultivation time and maximum fresh cell density was achieved at the stationary phase. The specific bromoperoxidase activity decreased during the exponential phase of growth and then increased considerably during the late exponential and stationary phases of growth, suggesting that bromoperoxidase production is a part of the secondary metabolism. The application of a photobioreactor is an appreciated development in the field of marine bioprocessing. The advantages of this type of reactor are that they offer cultivation under a wide variety of conditions and prevent contamination of the production strain by undesirable microorganisms. Benefits also include higher areal productivities and the prevention of water loss by evaporation. On the other hand, even if areal and volumetric productivity are higher than in open ponds, the performance does not come close to theoretical maxima and cannot even reach values obtained at lab scale. Moreover, the lack of performance, investment, and operation costs are still estimated as being far too high. It is expected that further research will enhance bioreactor performance and reduce capital costs [20].

Three US patents [21–23] related to the halogenation of cephalosporins have been granted. A starting cephalosporin, cephalexin, was incubated under appropriate reaction conditions with an enzyme preparation named cephalexin haloperoxidase. This enzyme preparation was obtained from *Rathayibacter biopuresis*, a unique bacterium of the *Rathayibacter* genus (of the order Actinomycetales), found in the gut of the marine worm *Notomastus lobatus*. This enzyme preparation enzymatically converts cephalexin to cefaclor. The haloperoxidase enzyme preparation described in the inventions is in the form of a crude extract from the microorganism produced through an inexpensive batch process. While this is a definite advantage, batch to batch variability of haloperoxidase activity may affect overall process performance.

#### 2.1.6. Amylase

Enzymes that are capable of digesting ungelatinized potato starch granules are economically attractive because they can enhance the range of potential starch sources. The marine yeast, *Aureobasidium pullulans* was found to produce more extracellular amylase than any other strains tested [24]. The crude glucoamylase production by the marine yeast and its potato starch granules digesting activity were investigated, aiming at exploring its probable applications in starch processing. The cultivation was carried out with different aeration rates of 2.0, 4.0, 6.0 and 8.0 L/min and various agitation speeds of 150, 200, 250 and 300 rpm, respectively. Highest yield of amylase was produced when the cell growth reached the late stationary phase. Although the study on the production of amylase by marine yeast is an interesting development, the described process suffers from improper optimization of the cultivation parameters. The production time in the bioreactor in comparison to shake flask cultivations was not reduced, which is a major drawback of process optimization. In the absence of data on dissolved oxygen profiles, it is difficult to conclude if aeration rate of 6 L/min and agitation speed of 250 rpm were really optimal.

#### 2.1.7. Multiple enzymatic activities

The bioprocessing parameters (agitation, aeration, and pH) were optimized for the growth and production of mycolytic enzymes (chitinases, proteases and glucanase) by a novel marine isolate *Pantoea dispersa* [25]. A one-factor-at-a-time approach was used in which all factors were kept constant except one. Thus, agitation (100–600 rpm) was optimized first, followed by aeration (0.25–1.5 L/L/min) and pH (6.8–8.8). During the optimization of aeration and agitation, the dissolved oxygen concentration and pH of the medium were initially adjusted to 100% air saturation and 7.2, respectively, and were not further controlled throughout the cultivation. However, changes in DO concentration and pH were monitored at 12 hours intervals. The advantage of the system is that it provides less expensive and efficient method to obtain high level of mycolytic enzymes of *P. dispersa*. Utlization of chitinous wastes not only solves an environmental problem but also decreases the cost of production of mycolytic enzymes. The formulated product can be used in the fields to protect crops against fungal diseases. However, direct use of the waste as raw material in the bioreactor might require longer sterilization times and create batch to batch variability. Weiner *et al*. [26] isolated the *Saccharophagus degradans* strain 2-40 from decaying *Spartina alterniflora*, a salt marsh cord grass in the Chesapeake Bay watershed, USA. *S. degradans* strain 2-40 was able to degrade many complex polysaccharides, including cellulose, pectin, xylan, and chitin, algal cell wall components, such as agar, agarose, and laminarin, as well as protein, starch, pullulan, and alginic acid. In addition to degrading this wide range of polymers, *S. degradans* strain 2-40 could utilize each of the polysaccharides as the sole carbon source. Therefore, *S. degradans* strain 2-40 is not only an excellent model for microbial degradation of insoluble complex polysaccharides (ICPs: polymerized saccharides that are used for form and structure in animals and plants) but can also be used as a model for complete metabolism of these ICPs. The lag phase was for 4 hours, the log phase lasted 16 hours with generation time of 2.3 hours. The stationary phase was reached after 20 hours and lasted for 12 hours after which the decline phase began. The most productive time for harvest is the beginning of the decline phase. The central highlight of this process is selection of the Strain 2-40 which has very high turnover rates.

#### 2.1.8. Superoxide dismutase

Superoxide dismutase (SOD) is a metalloenzyme that catalyzes the dismutation of superoxide radicals and has several applications in medicine. *Debaryomyces hansenii* strain C-11 was isolated from the Pacific Ocean off the west coast of Baja California Sur, Mexico [27]. The physical variables for biomass production were optimized and the effects of inducers (100% oxygen or 0.8 mM copper sulfate) for the enzyme were studied. The stirring rate was a variable of major importance in yeast growth. The quantity of air supplied to the reactor is an important factor, given *D. hansenii* needs oxygen for growth. When the airflow was excessive, the observed effects were the same as noticed with a fast stirring rate: bubble and foam formation. *D. hansenii* has an exceptional ability to grow under anaerobic conditions, although the authors’ results were not in agreement with this. They reported that when air is not supplied, the yeast grows only slightly, which may be just a consequence of the stirring rate allowing the cells to utilize whatever oxygen is present in the reactor. The SOD activity increased by application of inducers, pulse of oxygen and 0.8 mM copper sulfate into the bioreactor. The SOD activity varied throughout the growth cycle of *D. hansenii* at optimum conditions. At the beginning, SOD activity was low during the lag phase, but during the log phase, when the quantity of cells increased, the demand for oxygen and the production of free radicals were also at their maximum. Thus, the maximum SOD activity was found in the logarithmic growth phase. The authors established this organism as a good alternative source for SOD with a high specific activity. The cell biomass yield of this yeast was comparable to that reported for similar organisms but it possessed an interesting ability to grow in either a seawater or freshwater medium, which added some leverage in reducing contamination risks. However, spiking the reactor contents with pulse of 0.8 mM copper sulfate for 30 min would require process automation which may increase the final product cost.

#### 2.1.9. Other enzymes

For production of pyrophosphatase, Slater *et al*. [28] cultivated *Thermus thermophilus* in anaerobic culture The cells were grown to early stationary phase, after which they were harvested for pyrophosphatase. The high costs involved in maintenance of high process temperature as well as anaerobic conditions will add to the final product cost, which is a disadvantage of the process.

Esterase from the marine invertebrate, *Plexaura homomalla* was produced by two steps, first, extracting colonies or colony pieces of the animal with liquid acetone for a sufficient time to remove substantially all soluble lipids and second, recovering the acetone-insoluble matter. Contact with acetone was continued until substantially all of the soluble lipids were removed. The end-point was determined simply by examination of the acetone as by evaporation and by physical measurements on any residue obtained. The extraction temperature was kept below 50 °C to avoid denaturation of the enzyme, and was preferably in the range 20 °C to 30 °C. Lower temperatures may be used but the extraction would then proceed more slowly. The extraction is generally done at atmospheric pressure, but it may be carried out at higher or lower pressures provided the acetone is in a liquid state when contacting the *Plexaura homomalla* [29]. This is certainly an inexpensive and convenient method, but the source of the raw material is problematic as large scale processing by this method will lead to severe loss of biodiversity of this member of coral reef communities.

Biomineralization, biosilicification in particular (*i.e.*, the formation of biogenic silica, SiO_2_), has become an exciting source of inspiration for the development of novel bionic approaches following “nature as model”. Siliceous sponges are unique among silica forming organisms in their ability to catalyze silica formation using a specific enzyme, silicatein. The 3D-primmorph culture system of *Suberites domuncula* [30] is a suitable model system to study spiculogenesis; primmorphs may also be used as for the production of tailor-made silicas under controlled conditions following a cell/tissue factory approach. Primmorphs are cell aggregates that are formed from sponge single cells by the addition of Ca^2+^ ions that have the capacity to proliferate and differentiate. Totipotent archaeocytes differentiate to somatic cells, including spicule-forming sclerocytes. They were then transferred into culture chamber slides and cultivated further in the aquarium in artificial seawater together with other sponges. The sponges were fed with 4 mL of phytoplankton twice a week and the seawater was supplemented with vitamins and trace elements twice a month. The mollusc, *Trunculariopsis trunculus* present in all specimens of *S. domuncula* were fed with 5 g krill and animal plankton every three days. The hermit crab *Pagurites oculatus* was also associated with the mollusc and sponge. Under these conditions most of the primmorphs attached to the culture dishes and started to rearrange their cells to a higher organization state. In the aquarium the primorphs formed new monaxonial spicules and styles surrounded by organic matrix material. Formation of canals and dermal membrane development was seen after three weeks of cultivation. This advantage of cell cultures is that they are completely controlled and can be manipulated for optimal production of target metabolites. The disadvantage of this process is a complicated method of nutrient supply to the organism. Also, the effect of growth limiting factors such as light and oxygen were not studied by the authors.

Algae of the order Ulvales were modified to express genes encoding for alcohol dehydrogenase (ADH) or pyruvate decarboxylase (PDC) under the control of a heterologous high expression level promoter, such as the promoter of the algal ribulose bisphosphate carboxylase small subunit gene (SSU) or the algal pyruvate kinase gene as described in the US Patent by Moll [31]. The use of algae for producing soluble metabolic products has many advantages. The algae do not need to be harvested from growth ponds, nor do they need to be stirred, thereby greatly reducing pond costs. Algal systems also have a potential for very high productivity, since they have no stems or roots requiring metabolic support. Purification of product from the water phase is preferably accomplished by distillation or membrane separation. Algal systems tolerant to saline conditions are used so that seawater or brackish water can be used in irrigation of areas by the batch process, e.g., desert areas, utilizing a very low cost land. The advantage of this process is the low cost of operation but open-pond cultivation makes the process vulnerable to contamination as well as evaporative losses.

The respiratory chain system of a deep-sea barophilic bacterium, *Shewanella* sp. strain DB-172F (obtained from the Izu-Bonin trench at a depth of 6,500 m) was investigated by Quereshi *et al*. [32]. A membrane-bound ccb-type quinol oxidase was obtained from cells grown in a pressurized vessel. The bacterium was cultivated in pre-autoclaved bags containing Marine Broth 2216 in presence as well as absence of oxygenated fluorinert. The bags containing the medium were placed in titanium pressure vessels (manufactured by HiP, http://www.highpressure.com) and kept at atmospheric pressure (0.1 MPa) or pressurized at 60 MPa. This enzyme was specifically induced under conditions of elevated hydrostatic pressure and high levels were expressed in cells grown at 60 MPa. Results suggested the presence of two kinds of respiratory chains regulated in response to pressure in the deep-sea bacterium DB-172F. The difficulty of cultivation using high pressure vessels as well as increased operational risks would hinder translation of this otherwise very innovative cultivation technique into a commercial reality.

### 2.2. Immobilized processes

Immobilization of microorganisms for enzyme production offers various advantages, such as decreasing contaminants from the product stream during continuous cultivations without loss of biomass. Immobilized cells also prevent cell wash-out in continuous operation and allow increased productivity as well as operational stability. Marine bacteria are adapted to live attached to submerged surfaces, which often results in biofouling, but at the same time this unwanted characteristic is advantageous for immobilized cell processes.

#### 2.2.1. Proteases

**Figure 2:**
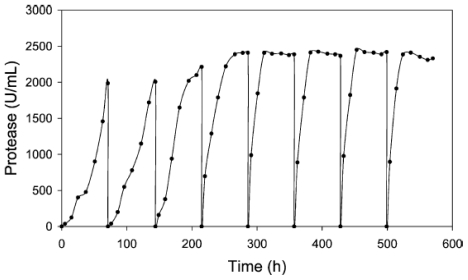
Repeated batch cultivation of *T. turnirae* at 3% sodium alginate, 3% CaCl_2_, 0.5 cell/alginate ratio and 200 beads. Reproduced with publisher’s permission from [33].

It has been reported that the marine shipworm bacterium *Teredinobacter turnirae* produces proteolytic activity of which more than 80% is extracellular. A particularly beneficial characteristic of the protease is its stability in the presence of oxidizing agents, absence of which is a major drawback with other proteases. Moreover, the protease produced is stable from 0 °C to 60 °C (with an optimum activity at 50 °C), at high salt concentration (3 M NaCl) and over a wide pH range (5–12). While the extracellular protease enzyme produced by *T. turnirae* has industrial potential, two negative factors have impeded its commercialization, first, the protease yield in the cultivation broth is relatively low and second, the bacterium is obligatory marine. Cell immobilization could be an effective alternative to grow the native bacterium and increase enzyme production. Elibol and Moreira [33] immobilized whole cells in calcium alginate beads which were used to produce alkaline protease. The objective of the study was to determine cultivation conditions for protease production by immobilized *T. turnirae* and this represented the first report on protease production by *T. turnirae* cells immobilized within calcium alginate beads. The cell suspension was aseptically added to sterile sodium alginate solution to achieve the required cell/alginate ratio. The mixture obtained was then extruded dropwise into CaCl_2_ solution and hardened in this solution. The beads were washed twice with sterile distilled water before being used as inoculum for protease production. The beads were used for eight successive batches each lasting 72 hours. It was also observed that there was a 3.5-fold increase in volumetric productivity of protease after the fourth cycle. The cycle time of the repeated batch culture was set to three days when the spent medium was removed and replaced with fresh medium. Protease production increased as the number of cyclic cultivations increased and reached a maximum after three cycles with a concomitant decrease in process time and an overall 3.5 fold increase in volumetric productivity (Figure 2). This is clearly an advantage of the process. However, the problem of diffusional limitation of mass transfer in the immobilized system needs to be further investigated.

In the next study by Beshay and Moreira, [34] the authors chose optimal conditions for immobilization of *T. turnirae* cells on different inorganic matrices and evaluated the immobilized biocatalysts in repeated batch cultivation for production of the novel alkaline protease. Immobilization experiments were carried out in 250 mL Erlenmeyer flasks. Alkaline protease activity produced by immobilized whole cells was about 2.3 times higher than that produced by freely suspended cells under the same cultivation conditions. The activity of alkaline protease produced by immobilized cells increased gradually and reached a steady state after 4 cycles. In another study by Beshay and Moreira, [35] *Teredinobacter turnirae* cells were adsorbed onto different matrices namely, ceramic support, broken pumice stone and silicone foam. These matrices effectively retained biomass and increased volumetric productivity by over 207% when compared to free cells. Enzyme productivity by immobilized cells in ceramic support and silicone foam were about 2.1-fold higher than the corresponding free cells. Repeated batch production of alkaline protease by immobilized cells in silicone foam matrix was achieved in five repeated batches without a significant decrease in the production of protease enzyme. In these studies, application of carriers of different porosities led to gross improvement in overcoming problems related to diffusional limitation of mass transfer and overall volumetric productivity increased as well. However, the prospects of scale-up to industrial levels remained unclear.

#### 2.2.2. Peroxidase

A superior method of producing extracellular products from aerobic microorganisms, particularly for producing lignin peroxidase from the marine fungus *Caldariomyces fumago* was developed [36]. In the method described in the US patent, the microorganism was grown on one side of an oxygen-permeable surface in an aqueous medium while the opposite side of the surface was supplied with oxygen. The inventors found that by immobilizing the organism on an oxygen-permeable surface and by supplying oxygen to the organism through the oxygen-permeable surface, the production of the extracellular enzyme by the microorganism reached the highest level in over eight batch production periods. When the organism was suspended and air or oxygen supply to the organism was provided by being bubbled through the aqueous medium, decreased activity of lignin peroxidase was obtained. When the organisms were attached to an oxygen permeable membrane but oxygen was supplied by flushing it through the headspace of the reactor and not through the membrane, no enzyme activity was observed after the initial addition of production medium. It is necessary to stress the organism (*C. fumago*) in order to stimulate the production of peroxidase. Typically this is accomplished by reducing the supply of nutrients to the organism. When the nutrient supply is reduced and the fungus begins to starve, it initiates the production of lignin peroxidase from some of its own protoplasm. After a period of lignin peroxidase production, it is necessary to again feed the fungus and stimulate its growth. Thus, production typically proceeds through alternating cycles of growth and peroxidase production which are controlled by supplying the fungus with a growth-producing medium followed by an enzyme-producing medium which is deficient in certain nutrients. After a number of cycles, the microorganism may begin to show signs of exhaustion and its rate of enzyme production may fall. Rejuvenation of the microorganism can be accomplished by hosing down the membrane to remove older fungi and permit the growth of the younger fungi. This complicated physiology may be a hindrance to process scale-up to large scale production systems.

### 2.3. Solid-state processes

The term solid-state fermentation (SSF) denotes cultivation of microorganisms on solid, moist substrates in the absence of a free aqueous phase; that is, at average water activities (defined as the relative humidity of the gaseous phase in equilibrium with the moist solid) significantly below 1.0. In a broader definition, SSF can be seen as including processes during which microorganisms are cultivated in the presence of a liquid phase at maximal substrate concentrations or on inert carriers. Although an aerobic process, the word “fermentation” has been long associated with solid-state processes due to use of this word in the biochemical industry. SSF technology has been known for centuries; from approximately 2,600 BC, it was used by the Egyptians for making bread, and information on the “koji process” dates back to 1,000 BC. Koji is an enzyme preparation produced by growing fungi such as *Aspergillus oryzae* on steamed rice or other cereals and it is still used as a starter culture in the soy-sauce industry and in the production of many oriental foods. The koji process is of great historical importance to modern SSF technology and can be considered as the prototype of SSF. Other SSF processes that have existed for centuries include: fermented foods (e.g., tempeh, miso and pozol); mold-ripened cheese (e.g., Roquefort); starter cultures for fermented brews; ensiling and composting. More-recent applications of SSF include the protein enrichment of agro-industrial residues, production of enzymes, organic acids and other fungal metabolites and spore production. Application of SSF for production of marine enzymes is also a recent development. This section has been categorized based on the nature of the solid support used, either organic and utilizable by the producing microorganism or inert and not assimilated by the cultivated organism [37,38].

#### 2.3.1. Use of organic media

l-Glutamine amidohydrolase is a potent antileukemic agent and a flavor-enhancing agent used in the food industry. *Vibrio costicola* isolated from the marine environment, produced extracellular l-glutaminase under solid-state process [39]. The suitability of different organic substrates was evaluated. Among the various solid substrates tested, wheat bran and rice husk favored maximal yield of enzyme compared to sawdust, copra cake powder, and groundnut cake powder. Enzyme yield was maximal using wheat bran and rice husk. Results indicated the scope for economic production of L-glutaminase by marine *Vibrio costicola* using solid-state process.

An alkalophilic and salt tolerant fungus *Beauveria bassiana* having chitinolytic activity was isolated from marine sediment and significant process parameters influencing chitinase production in solid-state process using wheat bran were optimized by Suresh and Chandrasekharan [40]. The organism was strongly alkalophilic. The NaCl and colloidal chitin requirements varied with the type of moistening medium used. Vegetative inoculum was more suitable than conidial inoculum for obtaining maximal enzyme activity and the addition of phosphate and yeast extract resulted in the enhancement of chitinase yield. This was the first report of the production of chitinase from a marine fungus.

Shellfish processing is one of the major agro-industries based on aquaculture, and has assumed great importance in recent years due to the ever-increasing demand for shrimps and crabs. The industry, however, is faced with the acute problem of disposing the alarming quantity of shellfish solid wastes. About 14%–27% of the dry weight of shrimp and 13%–15% of crab processing waste, comprise chitin depending upon the processing method. The conventional method of seafood processing includes chitin disposal by ocean dumping, incineration and land filling. However, factors such as cost of transportation and environmental pollution have prompted the search for alternative discarding methods. Bioconversion of waste is probably the most cost-effective and environment friendly procedure for waste utilization. The prawn waste can also be used as substrate for SSF. In the study by Suresh and Chandrasekaran [41], an attempt was made to utilize the solid waste from the prawn processing industry for chitinase production through SSF where the process parameters influencing SSF were optimized. Maximum chitinase yield was obtained in a medium containing a 5:1 ratio (w/v) of prawn waste/sea water after 5 days of incubation. The presence of yeast extract reduced chitinase yield. The results indicated scope for the utilization of shellfish processing (prawn) waste for the industrial production of chitinase by using solid-state process.

*Engyodontium album* an alkalophilic and salt tolerant fungus isolated from marine sediment produced protease, which was active at pH 11. Various process parameters influencing protease production by *E. album* were evaluated for maximal enzyme production using wheat bran as solid substrate and aged seawater incorporated with various nutrients as moistening medium [42]. The effect of each parameter on protease production was evaluated and then the significant parameters were optimized using statistical methods. Particle size of <425 mm, 60% initial moisture content and incubation at 25 °C for 120 hours were optimal for protease production using wheat bran. Sucrose as carbon source, ammonium hydrogen carbonate as additional inorganic nitrogen source and amino acid leucine enhanced enzyme production. Protease activity at high temperature and high alkaline pH suggested suitability of the enzyme for its application in the detergent industry.

Production of extracellular alkaline protease by a marine shipworm bacterium *Teredinocabacter turnirae* under solid-state process was optimized by Elibol and Moreira [43]. The maximum protease yield was achieved with optimized parameters such as coarse size of soybean, concentration of soybean, unadjusted pH of the medium, inoculum level and agitation rate that led to a 60% higher production. This study showed the feasibility of SSF for the first time for alkaline protease production employing the shipworm bacterium, *T. turnirae.*

Inulinase has received much attention as it can be widely applied to hydrolysis of inulin for production of fuel ethanol and high fructose syrup. Inulin is a linear β-(2,1)-linked fructose polymer that occurs as a reserve carbohydrate in Jerusalem artichoke, dahlia tubers or chicory root. Fructose is widely used in many foods and beverages instead of sucrose. Although inulin can be converted into fructose by chemical approach, this is associated with some drawbacks. Microbial inulinase yields 95% pure fructose after one step enzymatic hydrolysis of inulin. The central composite design (CCD), one of the response surface methodologies (RSMs) was used to optimize the medium compositions and cultivation conditions for the inulinase production by the marine yeast strain *Cryptococcus aureus* G7a in solid-state process [44]. Wheat bran and rice husk were used as the solid substrate. Under the optimized conditions, inulinase activity reached the predicted maximum activity derived from the RSM regression.

#### 2.3.2. Use of inert media

Natural substrates have a major disadvantage as the support material. The carbon source constitutes part of their structure. During the growth of the microorganism, the solid medium is degraded and as a result, the geometric and physical characteristics of the medium change, leading to channel formation, thus reducing heat and mass transfer. This limitation can be overcome by the use of an inert support with a more or less constant physical structure throughout the process. An additional benefit of SSF on inert supports is less complicated product recovery after completion of the process. The extracellular products with fewer impurities can be easily extracted from the inert support. Other advantages of SSF on inert support compared to SSF on natural organic substrates include reuse, the design of defined production media, application of mass balances for more advanced process modeling and control [38].

Polystyrene beads, impregnated with mineral salts/glutamine medium as inert support, were used to produce l-glutaminase from *Vibrio costicola* by solid-state process by Prabhu and Chandrasekaran [45]. Glucose enhanced the enzyme yield by 66%. The support system allowed glutaminase to be recovered with higher specific activity and lower viscosity than when a wheat-bran system was used. In another investigation by Prabhu and Chandrasekaran [46], the best process parameters influencing l-glutaminase production by marine *Vibrio costicola* in solid-state process using polystyrene as an inert support were selected. Maltose and potassium dihydrogen phosphate enhanced enzyme yield by 23% and 18%, respectively, while nitrogen sources had an inhibitory effect. As in the earlier study, leachate with high l-glutaminase specific activity and low viscosity was recovered. In the investigation by Sabu *et al*. [47], the potential of *Beauveria* sp. for l-glutaminase production using polystyrene as solid support under solid-state process was evaluated. This was the first report on extracellular enzyme production using inert support under solid-state process by any marine fungi. Enzyme production was growth associated.

#### 2.3.3. Advantages and disadvantages of solid-state processes

The aspects in favor of and in opposition to the processes described have been unified in this section. Solid-state process, an environmental-friendly bioprocess offers numerous advantages over submerged process for the production of bulk chemicals and enzymes such as simplified downstream operation, reduced energy requirements, lesser waste water produced, high yields of products, increased volumetric productivity, enhanced product recovery and simplicity of bioreactor design. This low-cost technology bioprocess is particularly suitable for the needs of developing countries.

The reasons against the application of SSF are engineering problems, the low compliance of the processes to standardization and the limited reproducibility of the results. Scale-up represents a particular problem because several different gradients (temperature, humidity, substrate concentration), which can arise in the course of the process, can have adverse effects especially in static solid bed processes and in processes involving substrate agitation. Inter-relationships among process parameters such as oxygen content, moisture level and temperature contribute to the difficult regulation of these variables. The microbial growth under aerobic conditions in the bioreactor results in a considerable production of heat that causes fast increase in temperature. This effect is often detrimental to bioprocesses because a large part of the enzymes produced during the cultivation can be heat denatured at the end of the process. In the absence of a free aqueous phase, the produced heat is difficult to remove. The cooling of the process takes place through evaporation, which requires very high aeration rates. The rising metabolic activity and the associated increased heat production have to be overcompensated by high aeration intensity. The water escaping by evaporation has to be in many cases replenished which can lead to undesirable local increase in water activity during static processing as well as local insufficient oxygen supply to the microorganisms. Substrate mixing may help but it should also be considered that many microorganisms are sensitive to the shear stress. Finally, the production of metabolic water by aerobic microorganisms can cause problems in the formation of conidiospores. It is expected that further research into the processes described is warranted to bring about improved monitoring of parameters, optimization and standardization [37].

## 3. Fed-batch Processes

Fed-batch cultivation is a production technique intermediate between batch and continuous processes. A proper feed rate, with the right composition is required during a fed-batch operation. Under regulated conditions and with the required knowledge of the microorganism involved in the cultivation, the feed of the required ingredients for growth and/or other substrates required for the production can never be depleted and the nutritional environment can be maintained fairly constant during the course of the batch. The production of by-products that are generally related to the presence of high concentrations of substrate can also be avoided by limiting its quantity to the amounts that are required solely for the production of the target compound. Sometimes, controlling the substrate is also important to overcome catabolic repression (inhibition of synthesis of enzymes involved in catabolism of carbon sources other than the preferred one). Since this method usually permits the extension of the operating time, high cell concentrations and thereby, improved productivity especially for the production of growth-associated products can be achieved. This method allows the replacement of water loss by evaporation and decrease of the viscosity of the broth. Fed-batch is a useful option for processes with low solubility substrates. If substrate is inhibitory, intermittent addition of the substrate will improve the productivity of the bioprocess by maintaining a low substrate concentration. With recombinant strains, fed-batch mode can ensure the presence of an antibiotic throughout the course of the cultivation, with the objective of maintaining an antibiotic-marked plasmid. In fed-batch processes, no special piece of equipment is required in addition to the one required for batch operation [48,49].

Similar to the previous section on batch processes, an outline of the operating conditions and inferences drawn is given in Table 2. Following this, five sub-sections each describing the enzyme considered for production through a fed-batch process have been presented along with the process strategy as well as an evaluation of each process.

### 3.1. Sulfite oxidase

**Figure 3:**
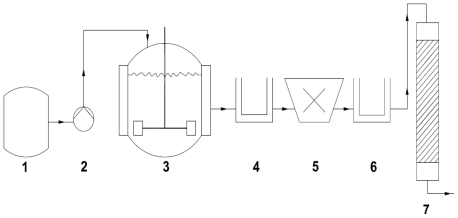
Fed batch cultivation for production of sulfite oxidase from *Sulfitobacter pontiacus* [50]. 1: Feed tank containing calcium sulfite solution 2: Pump 3: Bioreactor 4: Centrifuge for separating *S. pontiacus* cells 5: Sonicator for disintegrating cells for release of sulfite oxidase from *S. pontiacus* 6: Centrifuge for removal of cell debris from cell-free extracts 7: Sartobind S 100 membrane adsorber for purification of sulfite oxidase.

Sulfite oxidase catalyzes the oxidation of sulfite to sulfate and transfers electrons to oxygen, cytochrome c and a variety of other electron acceptors. A novel bioprocess to produce sulfite oxidase from the marine bacterium *Sulfitobacter pontiacus* was developed [50]. Preliminary marine broth cultivations indicated that the cells of *S. pontiacus* expressed only limited amounts of the sulfite oxidase when the sulfite concentration in the cultivation broth was low or absent. By supplementing a significant amount of sulfite to the cultivation medium a higher expression level of the enzyme could be induced. A high initial concentration of sulfite inhibited cell growth as well as enzyme expression and such temporary inhibitory concentrations of sulfite could be circumvented by the application of fed-batch processes. In a modified fed-batch approach, the substrate components in polyvinyl alcohol beads were immobilized for continuous release during the cultivation and the process compared to fed-batch cultivation in a stirred tank reactor. In the second part of the study, the authors concentrated on downstream processing which is the most cost-intensive component of the entire bioprocess. Therefore application of innovative techniques is crucial for process development to reduce investment as well as the subsequent operational costs. The use of membrane adsorber technology could be an advantage for such a development (Figure 3).

The modified fed-batch cultivation, which was performed in a process controlled bioreactor, resulted in a higher enzyme activity and cell density/biomass concentration. Furthermore, the generation time could be decreased by about 23% and the corresponding specific enzyme activity could be increased almost four times in the process. The application of a cationic ion exchange membrane adsorber demonstrated an excellent separation pattern and allowed an easy and fast purification of sulfite oxidase. The adsorber could be reused many times as well.

This is the first report of sulfite oxidase obtained from the marine bacterium *Sulfitobacter pontiacus*. The well-designed purification process could be used to supply increased amounts of the enzyme. As the membranes were used in a modular form, the process can be easily scaled up. A number of adsorbers can be assembled for process intensification. Although bioreactor cultivation has many advantages, compared to shake flasks, a transfer to other laboratories could be difficult due to the limited availability of bioreactors in many research laboratories. However, the easy handling of shake flasks and the negligible costs for providing such immobilized feed devices will allow application by many researchers. Additionally the use of immobilized feed technique reduces the contamination risk compared to sequential addition of sulfite to the medium and the necessary measurement of sulfite concentration in case of conventional cultivation. The main hindrance to cell growth in shake flasks is pH shift, which is easily controlled in a bioreactor by addition of acid. To circumvent this negative growth behavior and characteristics of enzyme expression a co-immobilization and release of buffering components should be realized in further experiments.

### 3.2. Protease

Beshay and Moreira [51] reported enhanced production of a novel alkaline protease from a newly isolated strain of *Teredinobacter turnirae* via fed-batch cultivation using a constant feeding strategy of C and/or N sources. Fed-batch cultivations were operated as batch processes for up to 24 hours, using either sucrose or NH_4_Cl or a mixture of them. Protease production started at the beginning of the growth phase at 24 hours, then the fed-batch cultivation was initiated and protease production continued to increase till 50 hours. The maximum protease activity was achieved after about 25 hours from starting the feeding process. Fed-batch culture was superior to batch culture of *T. turnirae* in producing alkaline protease. The maximum protease production rate of 158 U/mL/h was nearly 2.6-fold greater than values observed in batch operations.

### 3.3. Cellulase and xylanse

An US patent related to thermostable cellulase activity was awarded to Wicher *et al*. [52]. The polypeptides of the invention are variants of full-length or naturally occurring proteins that have thermostable cellulase activity and are readily produced in large quantities by expression in a host cell, *E. coli*. The polypeptide was derived from a thermophilic organism, *Rhodothermus marinus*, a thermophilic heterotrophic slightly halophilic marine eubacterium that grew optimally at 65 °C. *R. marinus* produced several thermostable glycosyl-hydrolases including a cellulase (Cel12A) [53]. Fed-batch cultivation for production of the Cel12A protein was carried out following Karlsson *et al*. [54]. A cultivation strategy for the production of two truncated thermostable recombinant xylanases (XynlAN and XynlANC) with *Escherichia coli* strain BL21 (DE3) as the host cell was described by the authors. The T7 *lac*-promoter used in this study allowed a strong and specific induction of the target sequence, but the presence of the T7 terminator hindered readthrough of the mRNA, which could lead to an overall decrease in productivity. As the cost of the inducer IPTG is a limitation in the development of a low-cost process, lactose was considered as an alternative inducer. A high xylanase production per unit cell mass was achieved through a fed-batch approach. Feed addition was observed by placing the feed vessel on a balance. The feed period was started upon initial substrate depletion, indicated by an increase in the DO signal or decrease in the stirrer speed. Feed was then added according to a predetermined exponential feed flow scheme as given in equation 1. Expression of the recombinant proteins was initiated by adding lactose (10 g/L) or IPTG (1 mM). The feed flow F(t) followed an exponential scheme as:

(1) $$\text{F}(\text{t})=\frac{X_{0}\;V_{0}}{Y(Sf-S)}\times \mu {\text{e}}^{\mu \tau }$$

where **X0** and **V0** are the cell dry weight (CDW) and volume at feed start, respectively, **Sf** is the glucose concentration in the feed, **S** denotes the glucose concentration in the bioreactor during the fed-batch phase, **Y** is the yield coefficient (gram CDW per gram glucose) and **μ**, the specific growth rate.

The advantage of this process was that a high xylanase production per cell was achieved through the fed-batch process. The low glucose level in the fed-batch phase allowed lactose induction with production efficiencies in the same range as IPTG induction. A potential shortcoming of the process is a complicated control mechanism which would require sensitive detectors to maintain the desired feed flow. The automated control will add to the final product cost.

### 3.4. Glutamate dehydrogenase

A process for producing a recombinant glutamate dehydrogenase protein by large scale fed-batch culture of a microbial transformant, while continuously controlling the concentration of dissolved oxygen and/or continuously adding assimilable sources, vitamins and nitrogen sources was patented [55]. *E. coli* JM109 was transformed with pTRP plasmid incorporating the recombinant glutamate dehydrogenase gene obtained from *Pyrococcos* sp. KODI and mass produced. Fresh medium in an amount corresponding to 10% to 15% of the initial culture medium volume was added at a progressively increased rate and the turbidity of the medium was monitored since the start of culture. The culture broth in the same amount as the amount of fresh culture medium was added, but not drained and thus the cell concentration of the medium gradually increased over time. The culture medium used in the fed-batch culture may have the same composition as that of a medium for ordinary batch culture. Alternatively, the culture medium may further contain assimilable sources such as saccharides, vitamins, nitrogen sources such as amino acids or other substances, which promote the growth of the cells being cultured and/or the expression of the desired gene. A nearly 5-fold increase in process productivity at completion of culture was achieved by the fed-batch culture in comparison to the batch process.

### 3.5. ADP ribosyl cyclase

**Figure 4:**
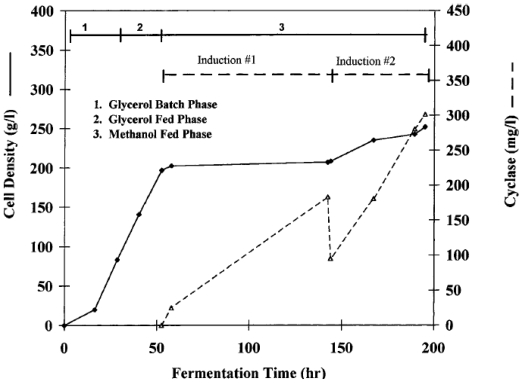
Production of cyclase through high biomass cultivation. Expression was induced with methanol when the culture had reached a cell density of 200 g/L. After about 100 h of induction and production of about 180 mg/L of cyclase, 2.0 liter of the culture was harvested, the yeast cells were re-suspended in fresh medium and induction was continued with higher methanol levels for an additional 53 h. Reproduced with publisher’s permission from [56].

Cyclic ADP-ribose (cADPR), a Ca^2+^mobilizing cyclic nucleotide derived from NAD^+^, is rapidly emerging as an endogenous modulator of Ca^2+^ induced release mechanisms in various cellular systems. ADP ribosyl cyclase, first isolated from the marine invertebrate *Aplysia californica*, cyclizes NAD^+^ to cADPR. Munshi *et al*. [56] utilized the methylotrophic yeast *Pichia pastoris* to express high levels of this enzyme. The protein was expressed using the tightly regulated methanol-inducible alcohol oxidase (*AOX1*) promoter and the *Saccharomyces cerevisiae* α-factor mating secretion signal. A bioreactor equipped with water-jacketed glass vessel and microprocessor controlled pH, dissolved oxygen, agitation, nutrient feed as well as foam control was used. Cultivation was carried out in three phases, the initial glycerol start-up phase, glycerol-fed phase, and the methanol induction phase (Figure 4). The advantage of the yeast system is the suitability for high-density growth contributing to increased production of the recombinant enzyme. The yeast expression system described in this process produced high level of expression of the cyclase as a secreted protein, which comprised about 95% of the total protein in the medium. This allowed a single-step process to purify the recombinant protein with high yields. The potential disadvantage would be the complex control systems required to carry out the process successfully. This would not only be expensive but also require considerable operator skill.

## 4. Continuous Processes

Continuous processes can perform better than batch processes by removing the intrinsic down time required for cleaning and sterilization and the lag phase before the organisms enter the period of high productivity. It is possible to maintain very high rates of product formation for long times with continuous cultivation. Interpretation of results is difficult for batch processes because concentrations of products and reactants, pH as well as redox potential change throughout the period of culture. The cells are also in different metabolic states: growing, active and dead cells. Continuous cultures exist in dynamic equilibrium and in uniform metabolic state. Considering industrial operation, capital and labor costs are reduced since there is only one process to manage. The production system can be tuned according to market demands; in peak periods the process can be run at a high flow rate, during lean periods the flow rates are reduced so that production rate is lowered. Although continuous culture achieves higher productivity from the reactor, there is no drastic improvement over batch culture in terms of the total number of equipment. Vessels will be necessary to prepare media and sterilization required to support the continuously operated system. Plant cleanliness requirements are more stringent than those required for batch process since a continuous system has the potential to run for a year or more during which time the vessels cannot be cleaned.

Depending upon the dynamics of the producer cells in the reactor, this section on continuous processes has been classified into two sections: suspended cell systems in which the cell is free within the liquid environment and immobilized systems in which movement of the cells is confined in a given space. Before discussing each process, Table 3 is presented providing an overview on the cultivation conditions and results obtained for the processes described.

### 4.1. Suspended cell systems

**Figure 5:**
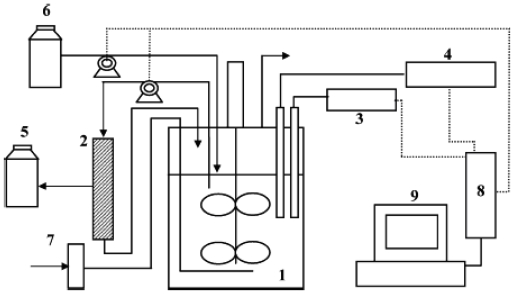
Experimental set-up for production of chitinase by *Paenibacillus* sp. CHE-N1. 1, bioreactor; 2, microfiltration module; 3, pH-meter and controller; 4, DO-meter; 5, permeate vessel; 6, fresh medium vessel; 7, air filter; 8, interface box; 9, computer. Reproduced with publisher’s permission from [57].

In the production of extracellular enzymes from bacteria, the removal of cells from culture medium is required in the purification process. Centrifugation and filtration techniques are generally used for this purpose. However, these operations are labor-intensive as well as time and energy consuming. Membrane technology provides a useful option for clarification of the spent medium. Ceramic membranes with proper mechanical, thermal and chemical properties and being resistant to the harsh cleaning treatments are considered a promising tool for developing microfiltration (MF) process for the treatment of the cultivation broth. In the study by Kao *et al*. [57], the performances of different ceramic membranes (cell retention, permeation flow rate, fouling and enzyme recovery) were evaluated under MF operation for the production of chitinase by *Paenibacillus* sp. CHE-N1. Batch and membrane experiments were performed. The external filtration unit used in this study was a Micro-CarbosepTM 40 module (Techsep, Miribel, France) equipped with 10 mm ID ceramic membrane column with different pore sizes, *i.e.*, M3 (15 kDa), M5 (10 kDa), M8 (50 kDa) and M9 (300 kDa), the total effective area of the membrane being 80 cm^2^. This filtration unit was coupled to the bioreactor. Membrane mode operations were carried out under the same conditions as in batch operations except for an outer loop MF module equipped with M9 membrane (which was found to be the most suitable) was added as can be seen in Figure 5. During the first 72 hours of the membrane mode operations, the cells were cultured batchwise, after that, the culture was switched to membrane mode operation with cell recycle. Deionized water was fed continuously to the bioreactor by a peristaltic pump at a volumetric flow rate of about 0.5–0.6 mL/min. The liquid contents were circulated through the filtration module under constant pressure by another peristaltic pump. The permeate was collected and the chitinase activity was analyzed. According to the proposed operation conditions, the chitinase activity could be stably maintained at a high level and harvested in a constant rate in the membrane mode operation with only DI water feeding along with the discrete chitin addition. The total chitinase activity obtained in membrane operation was about 78% higher than that obtained in batch mode operation. The continuous production of chitinase from *Paenibacillus* sp. coupled with membrane-based filtration might be considered as a suspended-cell culture under cell immobilization. Therefore, both the productivity and yield are higher than that obtained in batch culture, which can be considered as a major advantage of this system. The process can be improved by studying the deionized water replacement rate and the chitin supplementation conditions.

*Pyrococcus furiosus*, an obligate anaerobic heterotroph is a hyperthermophilic archaebacterium which grows optimally at 98–100 °C. When sulfur is present, H_2_S and CO_2_ are produced as a consequence of growth, along with trace amounts of H_2_. In the absence of sulfur, only CO_2_ is produced and the H_2_ eventually becomes inhibitory to cell growth. *P. furiosus* can reach cell densities of over 10^8^ cells/mL, which is relatively high for this class of organism, making it an attractive candidate for the production of enzymes for industrial applications. It has been found that *Pyrococcus furiosus* produces several starch hydrolyzing enzymes, both extracellularly and intracellularly which retain their activity for several hours at or above 100 °C. These comprise an α-glucosidase, which has been highly purified and amylolytic enzymes including an amylase and a pullulanase. In addition, another thermostable enzyme activity from *P. furiosus*, a β-glucosidase, useful in the process of degradation of cellulose to D-glucose has also been found, which is also active up to at least 95 °C.

A system that allowed continuous cultivation of hyperthermophilic archaebacteria at temperatures approaching 100 °C was developed. Using this system, continuous cultivation of *Pyrococcus furiosus* DSM 3638, was carried out and the resulting dilution rate and gas production profiles were determined [58]. The microbe was grown in artificial sea water supplemented with 0.1% yeast extract and 0.5% tryptone. Anaerobic conditions were achieved by flushing the medium with prepurified N_2_ and adding Na_2_S. For continuous culture, a 5-neck round bottom flask was designed as a culture vessel. A gas inlet tube was used to sparge the vessel and the gas stream exiting the reactor was passed through a Graham condenser to reduce water losses and then through a gas washing bottle containing 3.0 N NaOH to remove H_2_S. Samples for gas analysis were taken through a rubber septum mounted on the condenser outlet. The temperature in the culture vessel was maintained at 98 °C. Although *P. furiosus* grows optimally at 100 °C, operation slightly below this optimum prevented boiling, while supporting growth rates close to the maximum. Medium for continuous culture experiments was stored aseptically in sterile polycarbonate or polypropylene carboys and maintained under anaerobic conditions by purging prepurified N_2_. This culture medium was added to the reactor using a Masterflex peristaltic pump. A constant reactor volume was maintained using a dip tube and a size 16 pump head connected in parallel with the inlet pump. Approximately 10 mL of a late log phase culture was used to inoculate the reactor, which contained 750 mL of medium and 10 g/L elemental sulfur. The reactor was purged with prepurified N_2_ at a rate of 50 mL/min to ensure anaerobic conditions and mix the vessel contents. Continuous operation was initiated during the late log phase and the working volume of the reactor was maintained at 750 mL. Feed rate changes were made in the direction of increasing dilution rate and a minimum of three reactor volume changes were allowed after each adjustment for the system to reach steady state. The most significant result from this work was that cell densities approaching batch maxima could be achieved at relatively high dilution rates. Considering that these maximal cell densities are low in comparison with most mesophiles, it is was realized that the most efficient strategy for generating large amounts of *P. furiosus* biomass for production of enzymes would involve operating relatively small continuous reactors at high volumetric efficiencies. The yields per unit of culture medium and the reductions in labor and downtime for equipment cleaning of this approach compare quite favorably to the more usual batch approaches for cultivation of bacteria that grow only at lower temperatures. However, maintenance of anaerobic conditions may be problematic.

### 4.2. Immobilized cell systems

Sabu *et al*. [59] reported the continuous production of extracellular L-glutaminase by the marine fungus *B. bassiana* BTMF S-10 in a packed-bed reactor (PBR). Packed-bed reactors are the most widely studied bioreactor systems for immobilized cellular processes and most ideal when relatively longer retention times are required and external biomass buildup is minimal. Parameters optimized under batch mode for l-glutaminase production were incorporated into the continuous production studies. Calcium alginate beads with 12 × 10^8^ spores/g of beads were activated in a solution of 1% glutamine in seawater and the activated beads were packed into a glass column. The top of the bed was covered with a perforated Teflon disc. The medium was pumped using a peristaltic pump from the bottom and the effluent was collected from the top of the column. Air was introduced through a sparger at the bottom of the column after passing through a bacterial filter. Samples were collected at 1 hour intervals and assayed for enzyme activity. The effect of flow rate of the medium, substrate concentration, aeration rate and bed height on continuous production of l-glutaminase were investigated. Continuous production of the enzyme by Ca-alginate-immobilized spores resulted in a higher yield of enzyme within a shorter time. Scale-up may be difficult due to undesirable thermal gradients leading to poor temperature control and channel formation which occur in packed-bed reactors.

Kumar and Chandrasekaran [60] described the continuous production of l-glutaminase by Ca alginate immobilized cells of *Pseudomonas* sp. BTMS-51 in a PBR. A glass column reactor was used for the experiment. The reactor had a bottom support of perforated glass and activated beads were aseptically introduced into the reactor and packed. A perforated teflon disc was placed over the beads to prevent bed expansion during operation. Enzyme production medium was introduced from the top of the column and the effluent was collected from the bottom. The inlet and outlet flow rates were kept equal to maintain the liquid level constant at just above the bed level. The data obtained for 20 cycles of repeated batch operation indicated that immobilized cells were active and could be reused. The yield showed correlation with the biomass content in the beads, indicating that enzyme production was a function of cell concentration under the conditions of operation. It was observed that substrate depletion or product accumulation did not exert a dominant influence on enzyme production within the short time of retention employed in the cycles, and there was no reduction in the immobilized cell biomass after 20 cycles. The results showed potential for repeated use of immobilized cells without reduction in biomass and enzyme synthesis. The advantages of the process are first, the increase in yield that probably results from the greater availability of substrate per unit surface area of the microbial cells for inducing the synthetic machinery of the cells. Second, the system attained equilibrium between growth of the cells within the beads and their death and detachment from it. Such a steady state is particularly desirable in continuous processes employing immobilized cells, eliminating the need for incorporation of a regeneration phase. The process suffers from several drawbacks. First, with increase in dilution rate, the rate of substrate supplementation increases, product dilution is also a simultaneous occurrence, which is probably the reason for an observed decrease in activity with higher dilution rates. Second, the probable existence of diffusional effects in calcium alginate gels at higher dilution rates could also have contributed to the observed decrease in activity. Third, considering the availability of substrate as a key factor in enzyme production by the immobilized cells, a substrate concentration of 0.5%, did not allow the cells to attain their maximal rate of synthesis. Substrate limitation and increase in dilution rates above 1.06/h resulted in lower productivity.

Polar regions provide terrestrial and marine habitats for psychrophilic and psychrotrophic microorganisms. Cold adapted enzymes produced by these microbes have good potential for biotechnological applications because running processes at low temperatures saves energy, protects thermosensitive substances and reduces the risk of contamination by mesophilic microbial communities. Enzymes that are efficient catalysts at about 0 °C are characterized by a lower conformational stability of molecules and a higher flexibility of catalytic domains in comparison with their mesophilic counterparts. The extreme thermolability of cold-adapted proteins favors their easy and selective thermal inactivation, which is advantageous for certain uses. Cold-active β-galactosidases have recently been the subject of intensive studies because of potential applications in dairy industry and biotechnology. β-Galactosidases catalyse hydrolytic cleavage of lactose, one of the components of milk. Lactose intolerance, manifested as intestinal disorders resulting from ingestion of this disaccharide, is a relatively common clinical condition. Dairy products for those with lactose intolerance should be lactose free, which be achieved by enzymatic treatment with microbial β-galactosidases. The commercial preparations have previously been derived from mesophilic microorganisms, mainly from the yeast *Kluyveromyces lactis*, and therefore they are optimally active at elevated temperatures, although this process should be carried out in the cold, during shipping and storage of dairy products. Makowski *et al*. [61] focused on characterization of the immobilized recombinant enzyme (*Pseudoalteromonas* sp. 22b β-galactosidase was expressed in *E. coli* ER2566 host) and its application for hydrolysis of lactose at 4, 15 and 30 °C in continuous production systems. Chitosan was used as the matrix for enzyme immobilization and the prepared beads (1–1.5 mm in diameter) were separated by filtration through nylon membrane, suspended in a 0.1% solution of glutaraldehyde and incubated for 2 hours to activate amino groups of chitosan. The beads were then suspended in β-galactosidase solution (cell-free extract from the *E. coli* transformant), incubated for 24 hours at 4 °C and separated by filtration. The immobilized preparation was washed several times with 0.05M potassium phosphate buffer and kept at 4 °C. The shelf life at 4 °C of the Antarctic β-galactosidase immobilized on glutaraldehyde-treated chitosan exceeded 12 months. Its activity was maintained up to 40 days for continuous lactose hydrolysis in milk, conducted at 15 °C in a column reactor. The process has two major advantages: first, glucose, a strong noncompetitive inhibitor of the soluble Antarctic β-galactosidase does not inhibit the immobilized preparation. This considerably increases the technological value of immobilized Antarctic β-galactosidase. Second, the degree of lactose digestion reached 93% in the continuous system at an enzyme/substrate ratio of 30 U/g (lactose). Immobilized β-galactosidases of *K. lactis* and *Pseudoalteromonas* sp. TAE 79 (cold-adapted) were less efficient in this respect (lactose content was reduced by 70% and 53%, respectively). However, the incomplete recovery of enzyme activity was either due to disadvantageous conformational changes in protein molecules caused by formation of covalent bonds between enzyme and carrier or to diffusional limitations between the solid immobilized enzyme preparation and the substrate is a shortcoming. Electron microscopy confirmed the reason as changes in conformation of a part of the enzyme molecules linked to the matrix.

## 5. Shake Flask Cultivations

When designing an industrial cultivation medium, a large number of probable cultivation substrates are available for selection. Each of these medium components could potentially have a beneficial or detrimental effect on microorganism performance or on the medium cost as well as volumetric productivity. The only way to asses this is in a set of medium optimization experiments. There could be several strategies for medium optimization, for example, changing one medium component concentration at a time, factorial experiments, partial factorial experiments, response surface methodology or neural networks. Regardless of which medium optimization strategy is chosen, a large number of experiments are needed. It is only practical to conduct these experiments in shake flask culture, because a large number of flasks can fit on a rotary or orbital shaker.

The huge amount of data from shake flask cultures appearing in the literature raises several questions. How comparable are the results from shake flasks to scaled-up higher volumes? Could the results achieved in a stirred tank 10-L bioreactor be very different to those obtained from shake flask culture? Some researchers believe that as the pH is not controlled in shake flasks, as the oxygen transfer capabilities of the shake flask is poor, as considerable evaporation takes place during shake flask culture and as shake flask cultures lack adequate mixing, characterization of microorganism performance or optimizations conducted in shake flasks do not have much relevance. Further, the morphology of mycelial organism can be different in a shake flask when compared to the morphology in stirred tank reactors and this difference alone makes shake flask studies of little use. Opposed to these arguments, another group of scientists and engineers contend that there is no other way to carry out medium optimization apart from shake flasks simply because the number of experiments to be conducted is very large. They reason that the effect of the different medium components is relative and therefore the best medium in shake flask culture will also be the best medium in the stirred tank [62].

In this pre-final section of the review, advanced shake flask cultivations are summarized in a tabular form. Studies relating to optimization of medium components with the prospect of large scale processing have been included. Patent literature related to the occurrence of novel enzymatic activity has also been included.

More than half of the references cited in Table 4 relate to statistical design of experiments for optimization of medium components with the future prospect of large scale processing. Statistical experimental planning, factorial design and design of experiments investigates defined input factors to a converting system from which mostly common and well-defined output factors or responses are generated, such as product yield and productivity. Applied in this way, design of experiments is similar to mass balancing. However, the strength of the design is that it also demonstrates how interactions between the input factors influence the output responses. These interactions are often difficult to determine and interpret with other methods. Till date, most applications of experimental designs have concerned optimization of the composition of growth and production culture media. For majority of the media optimization applications, the final product yield or final concentration is the aim of the design experiments. The objective is to find the most favorable combination of nutrient factors to maximize the cellular productivity by supplying a well balanced composition of nutrients that enhances the maximum yield of the product molecule. [96].

Patent literature (including patent applications) related to the occurrence of novel enzymatic activity has also been included in Table 4. A patent enables the owner to exclude competitors from copying an invention. This exclusivity enables the costs of research and development, large scale manufacturing and product marketing to be recovered. As patents can be transferred in much the same way as other types of property, the patent owner, if unable to exploit the invention directly, can sell it to others. Patent protection is pursued when the financial return expected from the period of exclusivity justifies the cost of obtaining and maintaining a patent. When large amounts of time and money have been spent on research, development and new manufacturing equipment, these costs will need to be recovered over a period of time. A patent can provide the necessary protection and therefore a number of inventors prefer to obtain a patent on their invention performed in small scale (such as shake flask cultures) before proceeding to industrial scale operations.

## 6. Conclusions and Future Directions

This review provides a comprehensive overview on the various bioprocess strategies adopted for the cultivation of marine organisms for production of enzymes. A good understanding of the production process will provide ample opportunities for successful scale up to the industrial level. We believe this review will be useful to both discovery scientists as well as bioprocess engineers. Discovery scientists can have an impression on the various options available for scale-up of their lead product. The bioengineers can have knowledge about the various approaches available for the mass production of marine enzymes, their relative advantages and disadvantages and thus be able to select the best alternative.

Scientists and engineers are constantly striving for the development of novel reactors through optimization and conversion of current technologies having the potential to yield more efficient units for the production of marine derived biochemicals. As an example, Wright *et al*. [97] found the occurrence of barotolerant strains amongst marine microbes from surface waters. The findings are important from the standpoint of effective bioprocess engineering because in many reaction systems the application of pressure may lead to enhanced yields of target metabolites within the structure of the Le Chatelier’s principle, *i.e.*, high pressure will favor an equilibrium state that results in a negative volume change. Yan *et al*. [98] proposed the new concept of “niche-mimic bioreactors”, which essentially is the cultivation of the producing microbe in reactor conditions that mimic its ecological niche. Sarkar *et al*. [99–101] designed a rotating disc bioreactor (RDBR) to mimic the environmental conditions from where actinobacterial isolates were obtained, in order to elicit higher production of the antimicrobial compounds. The RDBR was operated at an ultra-low rotational speed of one revolution per day, which mimicked the intertidal estuarine habitat of these marine isolates, supported biofilm formation and production of antimicrobial metabolites. This review ends with the expectation that many more such innovative bioreactor technologies will emerge in the realm of marine enzyme bioprocess engineering.

## Figures and Tables

**Table 1:** Batch processes for marine enzyme production.

Enzyme and source	Operational scale and working volume	Process parameters				Carbon source(s)	Enzyme activity	Reference
		pH	Temperatu re (°C)	Aeration rate (L/L/min) and agitation rate (rpm)	Time			
***Batch submerged processes***								
Extracellular protease (*Vibrio harveyi*)	Applikon, (Holland) 1.5 L (reactor volume), 1.0 L (working volume)	7.2	30.0	0.5, 700	9 h	Peptone 5 g/L, yeast extract 1 g/L supplemented with skim milk	4.28 U/mg protein	[7]
Alkaline protease (*Bacillus clausii* No. 58)	Model KF5L, Kobiotech, Korea 5.0 L (reactor volume, 3.0 L (working volume)	9.6	42.0	1.5, 400	40 h	Casein, corn starch, 0.5% (w/v)	15,300 U/mL	[8]
Subtilisin, alkaline protease (*Bacillus* Strain TA39)	Model LH2000 10 L (working volume)	pH 7.6	4.0 and 25.0	NR	212.5 h	Bactopeptone 5 g/L, yeast extract 1 g/L	6.85 U/mL	[9]
Alkaline metalloproteases (*Hyphomonas jannaschiana*)	NR	7.6	37.0	NR	17 h	Carbohydrate based medium	NR	[10]
Chitinase (*Penicillium janthineflum* P9)	ADI 1020, Applikon (Holland) 3.0 L (reactor volume), 2.0 L (working volume)	4.0	28.0	1.5, 500	7 d	Colloidal chitin 15 g/L, corn steep liquor 0.5 g/L	686 U/L	[11]
Chitinase (*Paenibacillus* sp. CHE-N1)	BTF-A5L, Bio-Top Inc., Taiwan 5.0 L (reactor volume), 3.0 L (working volume), 10% preculture	7.0	34.3	3.0, 200	56 h	Colloidal chitin 5 g/L, peptone 1 g/L	11.8 U/mL	[12]
Chitinase (*Verticillium lecanii* F091)	5.0 L (reactor volume, STR), 3.0 L (working volume), 10% preculture and 30.0 L (reactor volume, airlift bioreactor), 15.0 L (working volume)	4.0	24.0	0.6, 150 and 0.9	6 d	Maltose 4.52%, marine peptone extract 1.79%, shrimp powder 0.4%, isolated soy protein 0.3%	18.2 mU/mL and 19.9 mU/mL	[13]
Thermostable DNA polymerase (*Thermotoga maritime*)	12.5 L (working volume)	6.8	30.0 then shift to 35.0	2.0	25.5 h	Glucose10 g/L	29.1 U/mg protein	[14]
Thermostable DNA polymerases (*Thermotoga neapolitana*)	Anaerobic process; 10.0 L (working volume) and 6.0 L (working volume)	NR	75.0 and 37.0	NR	28 h and 5 h	Yeast extract, 10% and Luria broth (LB) containing 10 μg/mL tetracycline	17 U/mL crude lysate	[16]
Agarase (*Vibrio sp.* Strain JT0107)	Marubishi Eng. Co., Tokyo 5.0 L (reactor volume), 3.0 L (working volume)	8.0	25.0	0.5, 350	10 h	Polypeptone 20 g/L, yeast extract 4 g/L, agar 4 g/L	625 U/L	[17]
Arylsulfatase (*Sphingomonas* sp. AS6330)	10.0 L (reactor volume)	7.0	30.0	1.0, 250	48 h	Sucrose 20 g/L	1620 (U/mL)	[18]
Bromoperoxidase (*Ochtodes secundiramea*)	Planar bubble column photobioreactor 5.0 L (reactor volume), 3.0 L (working volume)	8.0–8.5	22.0	0.71	70 d	Photoautotrophic	1.9 μmol MCD/g DCW/min	[19]
Cephalexin haloperoxidase (*Rathayibacter biopuresis*)	10.0 L (working volume, seed culture), inoculum size 1%–2% and 550.0 L (working volume, bioreactor)	8.5	28.0	NR	20 h and 25 h	L-malic acid 0.05%	NR	[21–23]
Glucoamylase (*Aureobasidium pullulans*)	B. Braun, Biostat B2 (Germany) 2.0 L (reactor volume), 1.8 L (working volume)	4.0	28	6.0, 250	56	Soluble starch 1 g/100 mL sea water	10 U/mL	[24]
Mycolytic enzymes (chitinases, proteases and glucanase) (*Pantoea dispersa*)	5.0 L (reactor volume, 2.0 L (working volume)	7.2	30.0	0.5, 300	72 h	Acid-swollen chitin, 5.0 g/L	10.04 ± 0.19 U/mL	[25]
Alginases (*Saccharophagus degradans*)	Microferm New Brunswick Scientific (USA) 14.0 L (reactor volume), 8.0 L (working volume), 2% inoculum	7.6	25.0	6.0, 400	36	Glucose, alginate, xylose	1690 alginase units	[26]
Superoxide dismutase (*Debaryomyces hansenii* Strain C- 11)	Microferm New Brunswick Scientific (USA) 2.0 L (reactor volume), 1.0 L (working volume), 1% inoculum	5–7	40.0	5.0–7.0	24.5	Glucose 2%	400 U/mg protein	[27]
T th pyrophosphatase (*Thermus thermophilus* 111)	CHEMAP Ltd. (Switzerland) 450.0 L (working volume)	NR	70.0	8.0, 4	18	Disodium succinic acid 5.0 g/L, calcium succinic acid 0.5 g/L	1760 U/mg protein	[28]
Esterase (*Plexaura homomalla*)	Extraction process, 30.0 L (working volume)	NR	20.0–30.0	Not required	1	Not required	NR	[29]
Silicatein (*Suberites domuncula*)	Aquarium volume 130.0 L	NR	16	NR	7 weeks	Sea animal feeds	NR	[30]
Alcohol dehydrogenase, pyruvate decarboxylase *(Ulvales)*	Open pond 1.0 hectare	NR	NR	Not required	15–20 d	Photoautotrophic	NR	[31]
Quinol oxidase (*Shewanella* sp. Strain DB-172F)	Pressurized vessel at 60 MPa	NR	4	NR	6 d	Peptone 5g/L, Yeast extract 1g/L	25.4 μmol/min/mg	[32]
***Batch immobilized processes***								
Alkaline protease (*Teredinobacter turnirae*)	250 mL Erlenmeyer flask	8.0	30.0	0, 120	100 h (SB) 500 h (RB)	Sucrose 5 g/L	2410 U/mL	[33]
Alkaline protease (*Teredinobacter turnirae*)	Immobilized process with glass carriers; 250 mL Erlenmeyer flask	8.0	30.0	0, 120	50 h (SB) 16 d (RB)	Sucrose 5 g/L	6605 U/mL	[34]
Lignin peroxidase (*Caldariomyces fumago*)	Immobilized process in silicone tubing; 2.5 L (reactor volume), 1.5 L (working volume)	NR	37.0	0. 25, 40	3 d	Dextrose	230 U/L	[36]
***Batch solid-state processes***								
L-glutaminase (*Vibrio costicola*)	250 mL Erlenmeyer flask	7.0	35.0	NR	24 h	L-glutamine	NR	[39]
Chitinase (*Beauveria bassiana*)	250 mL Erlenmeyer flask	9.2	28.0	NR	60 h	Chitin	246.6 U/g IDS	[40]
Chitinase (*Beauveria bassiana* BTMF S10)	Petri plates (86 mm diameter and 17 mm height)	9.5	27.0	NR	5 d	Prawn waste (23.08% chitin)	248.0 U/g IDS	[41]
Protease (*Engyodontium album* BTMFS10)	250 mL Erlenmeyer flask	5.0 and 10.0	25.0	NR	5 d	Sucrose 0.1 M	15, 912 U/g IDS	[42]
Alkaline protease (*Teredinocabcter turnirae*)	250 mL Erlenmeyer flask	7.34	30.0	0, 120	63 h	Soybean 1%	1950 U/mL	[43]
Inulinase (*Cryptococcus aureus* G7a)	250 mL Erlenmeyer flask	5.5	29.0	NR	5 d	Inulin 20.0 g/L	420.9 U/g IDS	[44]
L-glutaminase (*Vibrio costicola*)	Solid-state process on polystyrene beads, 250 mL Erlenmeyer flasks	7.0	37.0	NR	36 h	Glucose	88.0 U/g IDS	[45]
L-glutaminase (*Vibrio costicola*)	Solid-state process on polystyrene beads, 250 mL Erlenmeyer flasks	7.0	35.0	0, 150	24 h	L-glutamine 3 % w/v, maltose 1% w/v	196 U/g IDS	[46]
L-glutaminase (*Beauveria* sp.)	Solid-state process on polystyrene beads, 500 mL Erlenmeyer flasks	9	27.0	NR	96 h	L-glutamine 0.25 % w/v, D-glucose 0.5% w/v	49.89 U/mL	[47]

Abbreviations: NR: Not reported; STR: Stirred tank reactor; IPTG: Isopropyl β-D-1-thiogalactopyranoside; MCD: *Monochlorodimedone*; DCW: Dry cell weight; IDS: Initial dry substrate; SB: Single batch; RB: Repeated batch.

**Table 2:** Fed-batch processes for marine enzyme production.

Enzyme and source	Bioreactor scale and working volume	Process parameters				Carbon source(s)	Enzyme activity	Reference
		pH	Temperat ure (°C)	Aeration rate (L/L/min) and agitation rate (rpm)	Time			
Sulfite oxidase (*Sulfitobacter pontiacus*)	10.0 L (working volume)	7.3	26.0	0.4, 600	22 h	Peptone 5 g/L, yeast extract 1 g/L	9.2 U/mg protein	[50]
Alkaline protease (*Teredinobacter turnirae*)	2.0 L (working volume)	8.0	30.0	NR	50 h	Sucrose 8 g/L	8266 U/mL	[51]
Xylanase (*Escherichia coli* strain BL21DE3)	Chemoferm FLC- B-3 (Sweden) 3.0 L (reactor volume), 2.0 L (working volume)	7.0	37.0	DO maintained at 30% saturation	14 h	Glucose 10.8 g/L	245000 U/CDW	[54]
Glutamate dehydrogenase (*Pyrococcos* sp. KODI)	New Brunswick Scientific SF-116 (USA) 16.0 L (working volume)	7.4	37.0	15.0, 800	10 h	Glucose	300 U/mg protein	[55]
ADP ribosyl cyclase (*Aplysia californica*)	Bioflo III 5.0 L (reactor volume)	NR	28.0	1.14	200 h	Glycerol	300 mg/L	[56]

NR: not reported; DO: dissolved oxygen; CDW: Cell dry weight.

**Table 3:** Continuous processes for marine enzyme production.

Enzyme and source	Bioreactor scale and working volume	Process parameters				Carbon source(s)	Enzyme activity	Reference
		pH	Temperature (°C)	Aeration rate (L/L/min) and agitation rate (rpm)	Time			
Chitinase (*Paenibacillus* sp. CHE-N1)	Biotop BTF-A5L (Taiwan) 5.0 L (reactor volume), 2.0 L (working volume)	8.5	34.3	3, 200	132 h	Crab shell chitin powder 55.4 g/L	42,800 mU/mL	[57]
α-glucosidase (*Pyrococcus furiosus* DSM 3638)	Anaerobic, 5.0 L (reactor volume), 2.0 L (working volume)	6.8	98.0	NR	NR	Yeast extract 0.1%	NR	[58]
L-glutaminase (*Beauveria bassiana* BTMF S-10)	Immobilized process; PBR, glass column 2.3 cm radius, 20.0 cm height	9.0	27.0	No aeration	18 h	L-glutamine 0.25% w/v, D-glucose 0.5% w/v	4.048 U/mL/h	[59]
L-glutaminase (*Pseudomonas* sp. BTMS-51)	Immobilized process; PBR, ID 3.6 cm, height 45.0 cm	6.0	30.0	NR	120 h	L-glutamine 20 g/L, D-glucose 10 g/L	13.49 U/mL/h	[60]
β-galactosidase (*Pseudoalteromonas* sp. 22b)	Immobilized process; PBR with substrate recycling	7.6	15.0 and 4.0	NR	40 d	Lactose in milk	3.7 U/mg protein	[61]

NR: Not reported; PBR: Packed bed reactor; ID: Internal diameter.

**Table 4:** Overview of the shake flask cultivations applied for the production of enzymes from marine microbes.

Enzyme	Source	Objective of study	Results of study	Reference
Protease	*Bacillus subtilis*	Optimize conditions for deproteinization of crustacean wastes in the preparation of chitin	The metal chelator sensitive neutral protease activity was highest (20.2 U/mL) at pH 8.0 and 50 °C with casein as substrate.	[63]
Proteases	*Pyrococcus furiosus*	New thermostable enzymes and the use of these enzymes both in proteolysis as well as protein and polypeptide synthesis	The serine proteases retained enzymatic activity at 100 °C. It facilitated highly specific and efficient peptide synthesis even at high temperatures with high yields.	[64]
Alkaline protease	*Aureobasidium pullulans*	Optimization of medium and cultivation conditions	First report of alkaline protease production by marine yeast. Maximum production of the enzyme (623.1 U/mg protein; 7.2 U/mL) was obtained. It had the highest activity at pH 9.0 and 45 °C.	[65]
Protease	*Bacillus subtilis*	Optimization of physical factors affecting the production	The thermostable, organic solvent-tolerant protease was high (4042.4 U/mg) after optimization.	[66]
Protease	*Vibrio* sp.	Mathematical modeling leading to scale-up to industrial level	On industrial scale, potential of protease production by Vibrio anguillarum was highest with rainbow trout and squid peptones.	[67]
Chitinase	*Vibrio* sp. (FERM BP-5769)	Effect of medium constituents on enzyme production	The marine psychrophilic strain was induced by chitin and derivatives such as chitin glycol singly or in combination.	[68]
Chitinase	*Pantoea dispersa*	Effect of nineteen medium components on chitinase production optimized by Plackett–Burman design.	4.21-fold increase in chitinase production was observed in 22^nd^ medium of Plackett–Burman experimental design.	[69]
Chitinase	*Streptomyces* sp. DA11	Statistical Plackett–Burman design and Box–Henken response surface methodology to optimize medium components for enhancing chitinase activity	The chitinase activity and the maximum cell dry weight were 39.2-fold and 2.6-fold higher than that of the basic medium.	[70]
Chitinase	*Paenibacillus sabina*	Statistical optimisation of medium components	Biomass and pH played an important role in increasing chitinase production. 2.74 fold increase in chitinase production was achieved.	[71]
Enzymatic method for chitin extraction	Co-cultivation of *Lactococcus lactis* and *Teredinobacter turnirae*	Biological treatment of prawn waste for chitin production	Highest chitinase yield 95.5% was obtained when *T. turnirae* was first inoculated followed by *L. lactis*. This provided an environmentally friendly alternative.	[72]
Laccase	Unidentified basidiomycetous fungi	Decolourization of paper and pulp mills, textile, dye-making industries and alcohol distillery effluents	Isolate NIOCC #2a was efficient in decolorization of various colored effluents by producing laccase, which was active at pH 3.0, 6.0 and 60 °C in the presence of seawater.	[73]
Laccase, lignin peroxidase and manganese peroxidase	Unidentified basidiomycetous fungi	Effect of carbon and nitrogen sources on the production of lignin- degrading enzymes	The lignin–degrading enzymes and decolorization of effluent depended on the type of the nitrogen sources used.	[74]
Manganese- dependent peroxidase (MNP) and laccase	*Flavodon flavus*	Decolorization of molasses spent wash	Colour of molasses spent wash (MSW) was reduced by 80% and total phenolics, chemical oxygen demand were reduced by 50% with the strain. There was no role for manganese-dependent peroxidase in MSW.	[75]
Laccase, manganese peroxidase and lignin peroxidase	*Aspergillus sclerotiorum* CBMAI 849, *Cladosporium cladosporioides* CBMAI 857 and *Mucor racemosus* CBMAI 847	Enzyme production with different carbon sources and salinity conditions by using statistical experimental design.	First report of lignolytic enzymes from zygomycetes of *Mucor* genus. Highest values for lignin peroxidase, manganese peroxidase and laccase were obtained with the fungus *M. racemosus* CBMAI 847.	[76]
Manganese- dependent peroxidase, lignin peroxidase and Laccase	White-rot fungus *Flavodon flavus* (K1)	A process for removal of dyes using the lignin-modifying white-rot fungus	The salt tolerant fungi is better suited for treatment of industrial wastes as it can grow in half strength seawater.	[77]
Esterase	*Bacillus licheniformis* MP-2	Optimization of medium composition and cultural conditions by Plackett-Burman and Box-Henken design	The marine MP-2 esterase activity was improved from 258.8 U/mL to 318.2 U/mL and approached almost about 95% of the predicted value.	[78]
Esterase	*Staphylothermus*, *Pyrodictium*, *Archaeoglobus, Aquifex*, *M11TL*, *Thermococcus*, *Teredinibacter* and *Sulfolobus*	Enzyme production from native or recombinant host cells	Novel enzymes, active peptide fragments, analogs and derivatives were reported.	[79]
Lipase	*Pseudomonas* sp. (MSI057)	Optimization of culture conditions for psychrophilic alkaline lipase production	The optimum temperature and pH for the enzyme production was 30 °C and 9.0, respectively. The activity of purified enzyme was optimum at 37 °C and showed 80% activity at 20 °C though activity was decreased above 50 °C.	[80]
Amylase	*Halobacterium salinarum* MMD047	Optimization of medium composition and cultural conditions	Production was highest in minimal medium supplemented with 1% sucrose.	[81]
Endoglucanase	*Teredinobacter turnirae*	Effect of various carbon and nitrogen sources on Teredinobacter turnirae	Sucrose, ammonium phosphates and Triton X-100 enhanced of endoglucanase production. Combination of the components improved the production by 3.6 fold.	[82]
Cellulase	*Marinobacter* sp. (MSI032)	Optimization of medium composition and cultural conditions	1% maltose, 1% peptone and casein supported maximal production at 27 °C and pH 9.0.	[83]
Cellulase	*Rhodothermus marinus*	Methods for producing thermostable cellulases	Polypeptides from *Rhodothermus marinus* were expressed in *E. coli.*	[84]
Alginate Lyase	*Vibrio* sp. YKW-34	Optimization of culturing conditions and medium composition	After inducing, the activity of alginate lyase, reached 5 U/mL. Alginate, Laminaria powder acted as inducer whereas fucoidan, cellulose and glucose had negative effect on the alginate lyase production.	[85]
Glutaminase	*Providencia* sp.	Screening of about 400 marine isolates, biochemical identification tests, 16S rRNA sequencing and media optimization studies	Applying response surface methodology, glutaminase activity and specific activity 119 ± 0.12 U/L and 0.63 U/mg protein respectively were obtained from one *Providencia* sp.	[86]
Glutaminase	Recombinant *Escherichia coli*	Overexpression of salt tolerant L- glutaminase from *Micrococcus luteus*	*Escherichia coli* JM109 transformed with pKSGHE3-1 exhibited more than 190-fold higher glutaminase activity than *M. luteus* K-3 under optimal culture conditions.	[87]
Recombinant hexose oxidase	*Chondrus crispus, Iridophycus flaccidum* and *Euthora cristata*	Expression of recombinant hexose oxidase in *Pichia pastoris*	An isolated DNA fragment having hexose oxidsae activity was expressed in *E. coli* or *S. cerevisae* or *P. pastoris*	[88]
Phytase	Marine yeast *Kodamaea ohmeri*	Medium optimization by response surface methodology	9-fold enhancement in phytase activity (from 62.0 to 575.5 U/mL) was attained after optimization.	[89]
PUFA polyketide synthase	*Schizochytrium* (a Thraustochytrid marine microorganism)	Production of PUFA polyketide synthase	Methods of making and using the non-bacterial PUFA PKS systems were described.	[90,91]
Biofilm degrading enzymes	*Microbulbifer* sp.	Isolation of biofilm degrading enzymes	Method for preparing biofilm degrading, multiple specificity, hydrolytic enzyme mixtures which are specifically tailored to remove targeted biofilms.	[92]
Superoxide dismutase	*Photobacterium phosphoreum, Photobacterium leiognathi* and *Photobacterium sepia*	Production of superoxide dismutase containing iron.	Process for the production of superoxide dismutase extracted from marine bacterial strains was described.	[93]
Silicatein	*Suberites domuncula*	Use of highly-expressed and highly active recombinant silicatein	A method for the synthesis of amorphous silicone dioxide, silicones or other silicon (IV) or metal (IV) compounds or mixed polymers of these compounds by contacting a silicon substrate with a polypeptide or a metal complex of a polypeptide comprising a carbonic anhydrase domain was described.	[94]
DNA ligase	*Pyrococcus furiosus*	Isolation of thermostable DNA ligase.	DNA ligase that retained its activity from 85 °C to 100 °C was described.	[95]
